# Horizontal transfer of vertebrate vision gene *IRBP* into the chordate ancestor

**DOI:** 10.1073/pnas.2310390120

**Published:** 2023-08-14

**Authors:** Anthony K. Redmond, Aoife McLysaght

**Affiliations:** ^a^Smurfit Institute of Genetics, Trinity College Dublin, Dublin 2, Ireland

Interphotoreceptor retinoid-binding protein (IRBP) plays a key role in vertebrate vision by transporting retinoids between photoreceptor and retinal pigment epithelium cells in the eye. Kalluraya et al. (1) report that vertebrate IRBP arose via horizontal gene transfer (HGT) of a bacterial S41 peptidase into the vertebrate ancestor, followed by domain duplications and neofunctionalization. These events likely occurred >500 ma, concomitant with the emergence of the vertebrate eye (1), exemplifying functionally coopted HGT in animals.

**Fig. 1.**
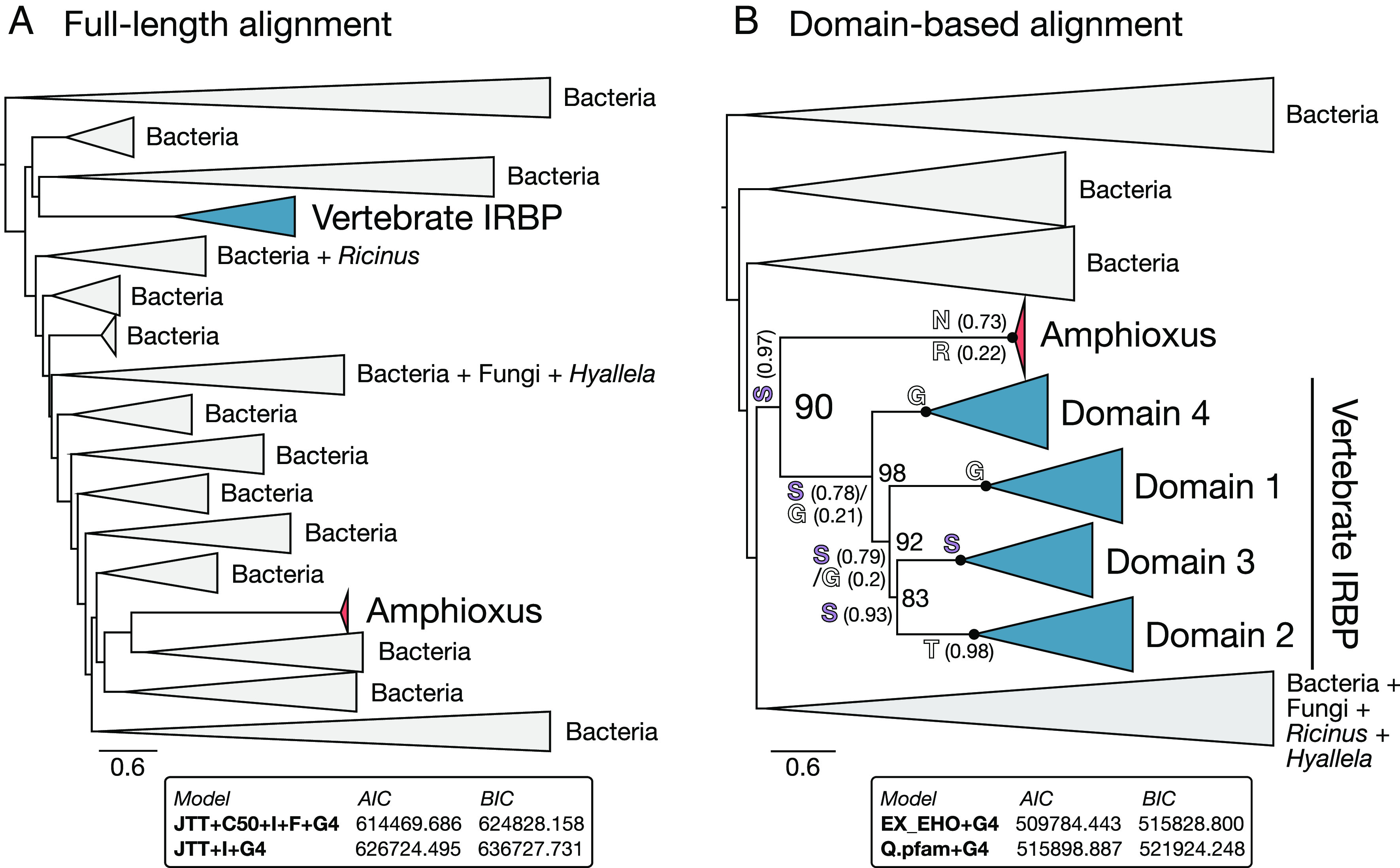
Phylogenetic analysis of vertebrate IRBP. IQ-TREE (version 2.2.0) (4) maximum likelihood analysis of the full-length alignment (Dataset S2 from ref. 1) (*A*) and domain-based alignment (Dataset S3 from ref. 1) (*B*) of Kalluraya et al. (1). Ultrafast bootstrap (5) percentages from 1,000 replicates are shown to the right of each node within Chordata for the domain-based alignment only (*B*), with black filled circles indicating 100% support for monophyly of each vertebrate domain and amphioxus sequences, and the larger font “90” representing 90% ultrafast bootstrap support for amphioxus homologs as sister to vertebrate IRBP domains. Branches are collapsed at major nodes and the tree rooted on the midpoint to aid visualisation for (*A*) and (*B*). IQ-TREE Ancestral state reconstruction of the active site serine position is shown for key nodes across Chordata (serine shown in purple; amino acids with posterior probabilities >0.05 shown, with nonmaximal posterior probabilities shown in parentheses), having been calculated using the maximum likelihood consensus tree as a fixed topology for the domain-based alignment only (*B*). The best-fitting models, site-heterogeneous JTT+C50+F+I+G4 (*A*) and EX_EHO+G4 (*B*), were identified using ModelFinder (6) in IQ-TREE. In each case the best-fitting model from Kalluraya et al was tested (shown in box at *Bottom*), as well as a suite of site-heterogeneous models [following (7)], including EX2 (8), EX3 (8), EHO (8), EX_EHO (8), UL2 (9), UL3 (9), C10, C20, C30, C40, C50, and C60 (10). C10 to C60 were also tested when paired with JTT (for *A* only) and Q.pfam (for *B* only) exchangeabilities based on best-fitting site-homogeneous model from Kalluraya et al. (1), while +F, +G4, +I, and their combinations were considered for all models. Full consensus tree files with ultrafast bootstrap supports are available on figshare (https://doi.org/10.6084/m9.figshare.23544741).

**Fig. 2.**
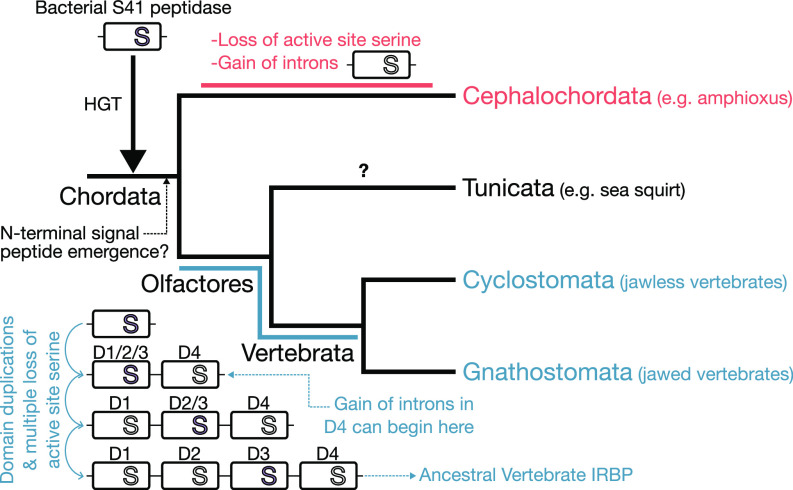
Proposed model of IRBP evolution in chordates. A bacterial S41 peptidase is horizontally transferred into the chordate ancestor and likely gains a signal peptide before divergence of Cephalochordata from Olfactores. Introns are gained and the active site serine is lost (purple = active, white = serine substituted as per Fig. 1) during cephalochordate evolution (red). After the split with cephalochordates, the proto-IRBP undergoes three domain duplication events, three separate losses of the active site serine, and accrual of introns in D4 (i.e., Domain 4; blue) prior to the divergence of extant vertebrates. Discovery of a tunicate IRBP homolog would help to refine this timing if the gene is not lost from this lineage.

Kalluraya et al. (1) propose independent HGTs into amphioxus and vertebrates, despite their close relationship within Chordata. However, they also noted difficulty in identifying the closest relatives to vertebrate IRBP and acknowledge that shared HGT is “difficult to completely rule out” (1). We performed new phylogenetic analyses of Kalluraya et al.’s full-length IRBP sequence alignment and their domain-based alignment using better-fitting site-heterogeneous models that can reduce phylogenetic error (Fig. 1). Reanalysis of the full-length sequence alignment still supports independent HGTs (Fig. 1*A*). However, domain-based reanalysis recovers the single-domain amphioxus sequences as sister to the vertebrate IRBP domains (Fig. 1*B*). This indicates that a single-domain S41 peptidase was transferred once into the chordate ancestor (Fig. 2), rather than separately into early vertebrates and amphioxus. The domain-based analysis should be more reliable as it considers IRBP’s full history (2), with the relationships of nonvertebrate sequences to all of the duplicate vertebrate domains being considered, rather than domain 4 alone (which all nonvertebrate sequences are aligned to in the full-length dataset). Furthermore, the domain duplications occurring prior to the divergence of vertebrates (1) (Fig. 1*B*) means that considering all domains breaks the long branch separating vertebrate IRBP from its homologs, which should reduce propensity for phylogenetic error (3).

Despite inferring a different HGT history, we propose a similar evolutionary scenario leading to vertebrate IRBP. First, monophyly of vertebrate IRBP domains (Fig. 1*B*) indicates the domain duplications occurred after the divergence of Cephalochordata (including amphioxus) and Olfactores (Vertebrata and Tunicata) (Fig. 2). Second, ancestral state reconstruction indicates independent loss of the active site serine in vertebrates (where it was likely lost three separate times) and amphioxus (Figs. 1*B* and 2). This suggests separate neofunctionalization in amphioxus, though testing this requires functional analysis (1). Third, we confirm independent emergence of introns in amphioxus and vertebrates (1). The presence of introns in only domain 4 of vertebrate IRBP (1) indicates that they emerged after the first domain duplication separating Domain 4 from the ancestor of Domains 1 to 3 (Figs. 1*B* and 2) and hence after the divergence with cephalochordates (Fig. 2). The separate occurrence of all major changes is consistent with the HGT event occurring shortly before Cephalochordates and Olfactores diverged, which could explain the weak signal for shared HGT when not considering all domains and best-fit phylogenetic models.

In summary, we propose a parsimonious single HGT of the IRBP progenitor into the chordate ancestor (Fig. 2), rather than two HGTs into closely related chordate lineages (1), refining our knowledge of the evolution of a key aspect of the vertebrate visual apparatus.
